# Protein 4.1N acts as a potential tumor suppressor linking PP1 to JNK-c-Jun pathway regulation in NSCLC

**DOI:** 10.18632/oncotarget.6312

**Published:** 2015-11-13

**Authors:** Zi Wang, Bianyin Ma, Hui Li, Xiaojuan Xiao, Weihua Zhou, Feng Liu, Bin Zhang, Min Zhu, Qin Yang, Yayue Zeng, Yang Sun, Shuming Sun, Yanpeng Wang, Yibin Zhang, Haibo Weng, Lixiang Chen, Mao Ye, Xiuli An, Jing Liu

**Affiliations:** ^1^ The State Key Laboratory of Medical Genetics and School of Life Sciences, Central South University, Changsha, China; ^2^ Department of Medicine, University of California, Irvine, CA, USA; ^3^ Department of Biochemistry, College of Medicine, Jishou University, Jishou, China; ^4^ Department of Histology and Embryology, Xiangya School of Medicine, Central South University, Changsha, China; ^5^ Molecular Science and Biomedicine Laboratory, State Key Laboratory for Chemo/Biosensing and Chemometrics, College of Biology, College of Chemistry and Chemical Engineering, Collaborative Innovation Center for Chemistry and Molecular Medicine, Hunan University, Changsha, China; ^6^ College of Life Sciences, Zhengzhou University, Zhengzhou, China; ^7^ Laboratory of Membrane Biology, New York Blood Center, New York, NY, USA

**Keywords:** protein 4.1N, non-small cell lung cancer, tumor suppressor, PP1, JNK/c-Jun pathway

## Abstract

Protein 4.1N is a member of protein 4.1 family and has been recognized as a potential tumor suppressor in solid tumors. Here, we aimed to investigate the role and mechanisms of 4.1N in non-small cell lung cancer (NSCLC). We confirmed that the expression level of 4.1N was inversely correlated with the metastatic properties of NSCLC cell lines and histological grade of clinical NSCLC tissues. Specific knockdown of 4.1N promoted tumor cell proliferation, migration and adhesion *in vitro*, and tumor growth and metastasis in mouse xenograft models. Furthermore, we identified PP1 as a novel 4.1N-interacting molecule, and the FERM domain of 4.1N mediated the interaction between 4.1N and PP1. Further, ectopic expression of 4.1N could inactivate JNK-c-Jun signaling pathway through enhancing PP1 activity and interaction between PP1 and p-JNK. Correspondingly, expression of potential downstream metastasis targets (ezrin and MMP9) and cell cycle targets (p53, p21 and p19) of JNK-c-Jun pathway were also regulated by 4.1N. Our data suggest that down-regulation of 4.1N expression is a critical step for NSCLC development and that repression of JNK-c-Jun signaling through PP1 is one of the key anti-tumor mechanisms of 4.1N.

## INTRODUCTION

Lung cancer is the most common malignant epithelial cancer and leads as cause of cancer mortality worldwide. NSCLC accounts for ∼80% of lung cancer cases and is characterized by a poor prognosis and resistance to antineoplastic drugs. Although significant therapeutic improvements have been made in recent years, NSCLC patients often relapse and develop metastases after surgery, resulting in a 5-year survival rate of less than 15% [1]. To improve the survival of NSCLC patients, the screening for growth and metastasis-related genes in lung cancer has significant value for prognosis and targeted therapy [2].

Protein 4.1N is a neuronal homolog of the erythrocyte membrane cytoskeletal protein 4.1. It serves as a critical component of the membrane skeleton and provides a connection between spectrin-actin networks and the transmembrane proteins. The mammalian 4.1N is enriched in central and peripheral neurons [3], where it is implicated in the stability and plasticity of the neuronal membrane, synaptic architecture and function, and surface localization and aggregation of receptors by associating its binding partners with the spectrin-actin skeleton [4–6]. 4.1N also exists in other organs, such as the heart, kidney, pancreas, lung, breast, ovary and large intestine [3]. To date, decreased expression of 4.1N has been reported to be closely associated with malignant potential in breast and ovarian cancer [7, 8]. Additionally, 4.1N has been shown to exert its anti-proliferative effects by antagonizing the role of NuMA in mitosis and inhibiting the activity of the PIKE (PI3K kinase enhancer) in PC12 cells [9, 10].

Protein phosphatase 1 (PP1) is a major eukaryotic phosphatase that regulates diverse cellular processes through dephosphorylating phospho-Serine/Threonine residues of proteins. PP1 catalytic subunits are composed of four isoforms, PP1α, PP1β/δ, PP1γ1 and PP1γ2, which are targeted by a variety of regulatory subunits that control catalytic specificity, activity and subcellular locations of PP1 isoforms [11]. PP1 is able to dephosphorylate/activate the tumor suppressor Rb (retinoblastoma protein), thereby maintaining cells in G1 phase and initiating apoptosis [12–14]. Furthermore, PP1 has emerged as a key regulator of cytoskeleton-associated processes. For example, PP1 dephosphorylates several actin-binding proteins, including myosin, merlin, and ERM (ezrin/radixin/moesin) family. These molecules are involved in organizing specialized membrane domains, such as microvilli, filopodia and lamellipodia, and membrane-cytoskeleton interactions [15–17].

In this study, we first analyzed the differential expression of 4.1N in NSCLC-derived cell lines with different metastatic potentials and in NSCLC tissues with different histological types and grades to determine if 4.1N was involved in the malignant progression of NSCLC. In addition, the anti-tumor properties of 4.1N were assessed *in vitro* and *in vivo.* Specifically, we identified that 4.1N interacts with PP1 and participates in the regulation of JNK-c-Jun signaling via the enhancement of the dephosphorylation of PP1.

## RESULTS

### Expression of protein 4.1N in NSCLC cell lines and NSCLC samples

**Figure 1:**
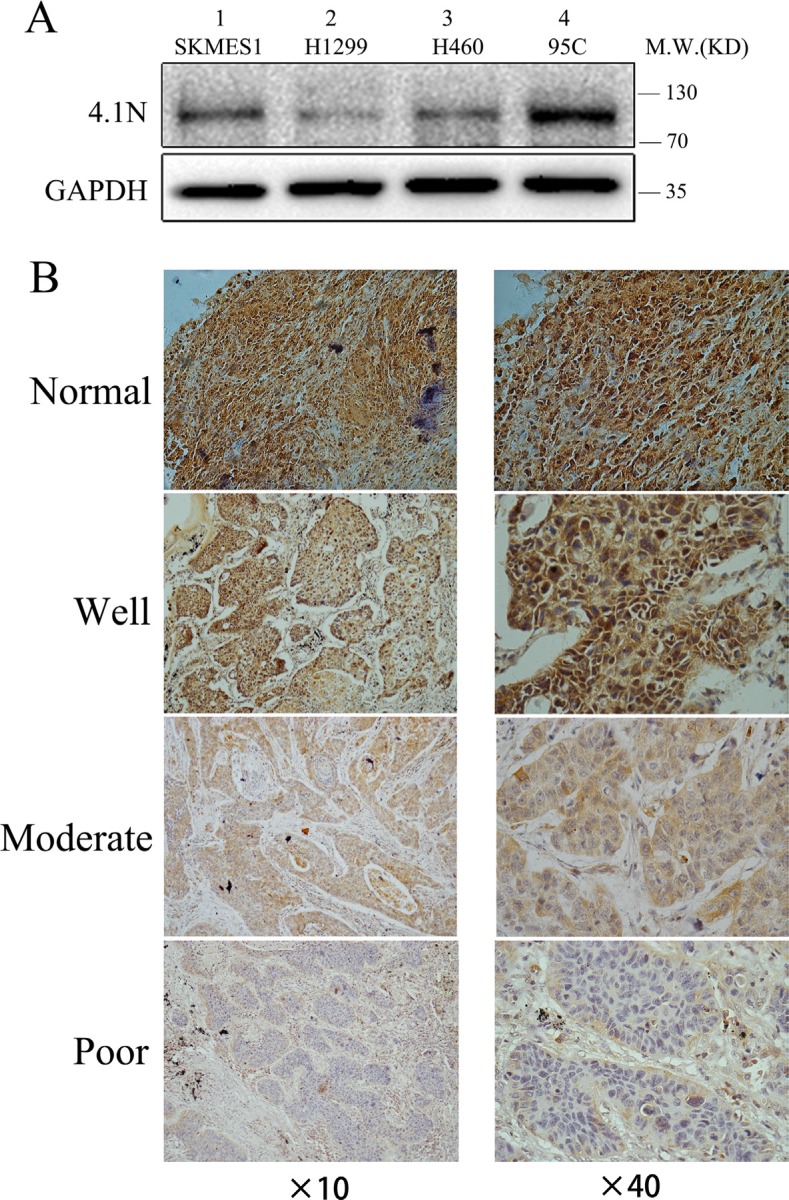
4.1N expression levels in NSCLC cell lines and primary tumors (**A**) Protein expression level of 4.1N was analyzed in four NSCLC cell lines with different metastatic abilities. 4.1N expression was decreased in metastatic tumor cell lines (lane 1–3) compared with low metastatic 95C cells (lane 4). (**B**) Representative images of immunohistochemical staining for 4.1N expression. NSCLC specimens and normal lung tissues were stained for 4.1N by an anti-4.1N HP antibody. The brown color represents positive staining for 4.1N expression. Cell nuclei were counterstained with hematoxylin (blue). The normal tissue showed an intense immunoreactivity for 4.1N. For tumor tissues, more intense staining for 4.1N was observed in well differentiated tumors compared with moderately and poorly differentiated tumors. Original magnification: × 40 and × 40.

To explore whether the expression levels of 4.1N were related to the progression of NSCLC, we first performed a Western blotting analysis to investigate the protein expression levels of 4.1N in four NSCLC cell lines with different metastatic potentials, including 95C (low metastatic potential), H1299 (derived from lymph node), H460 (derived from pleural effusion), and SK-MES-1 (derived from pleural effusion). In all tested cells, the apparent molecular weight of 4.1N was approximately 100 kDa, which represents a major splice variant of 4.1N in these NSCLC cell lines. Compared with 95C cell line, 4.1N protein expression level was markedly decreased in high metastatic potential H1299, H460 and SK-MES-1 cell lines (Figure 1A). The data suggest that the expression level of 4.1N was inversely related with the metastatic potential of NSCLC cell lines.

At the same time, we analyzed 4.1N expression levels by immunocytochemistry in 99 NSCLC specimens including 52 LAC (lung adenocarcinoma), 46 LSCC (lung squamous cell carcinoma) and 1 LCLC (large cell lung carcinoma), and in 10 normal lung tissues. The results showed positive staining for 4.1N in all normal lung tissues (10/10), whereas negative staining of 4.1N was detected in 52% (52/99) of NSCLC specimens including 55% (29/52) in LAC, 47% (22/46) in LSCC, and 100% (1/1) in LCLC compared with normal adjacent tissues (Figure 1B; Table 1; Supplementary Figure S1). In particular, negative staining of 4.1N was significantly associated with poorly differentiated cases and was more likely to occur in cases with a higher TNM stage (stage III/IV) (Table 1). Thus, loss of 4.1N was considered a relatively late event in NSCLC. Decreased expression of 4.1N potentially promoted tumor progression to advanced stages.

**Table 1:** Correlation of 4.1N expression with clinicopathologic features of patients with NSCLC

Variables	No. (*n* = 99)	4.1N expression		*p* value
		Negative (*n* = 52)	Positive (*n* = 47)	
Age (years)				
< 60	50	25(50%)	25 (50%)	0.611
≥ 60	49	27(55%)	22 (45%)	
Gender				
Male	66	33(50%)	33 (50%)	0.477
Female	33	19(58%)	14 (42%)	
Pathologic differentiation				
Well	12	2(17%)	10 (83%)	0.005
Moderately	33	15(45%)	18 (55%)	
Poorly	54	35(65%)	19 (35%)	
TNM stage				
I	29	10(35%)	19 (65%)	0.029
II	16	6(38%)	10 (62%)	
III	36	22(61%)	14 (39%)	
IV	18	13(72%)	5 (28%)	
Histologic type				
Squamous	46	22(48%)	24 (52%)	0.477
Adenoma	52	29(56%)	23 (44%)	
Large cell carcinoma	1	1(100%)	0	

### 4.1N suppresses the growth, migration and adhesion of NSCLC cell lines *in vitro*

**Figure 2:**
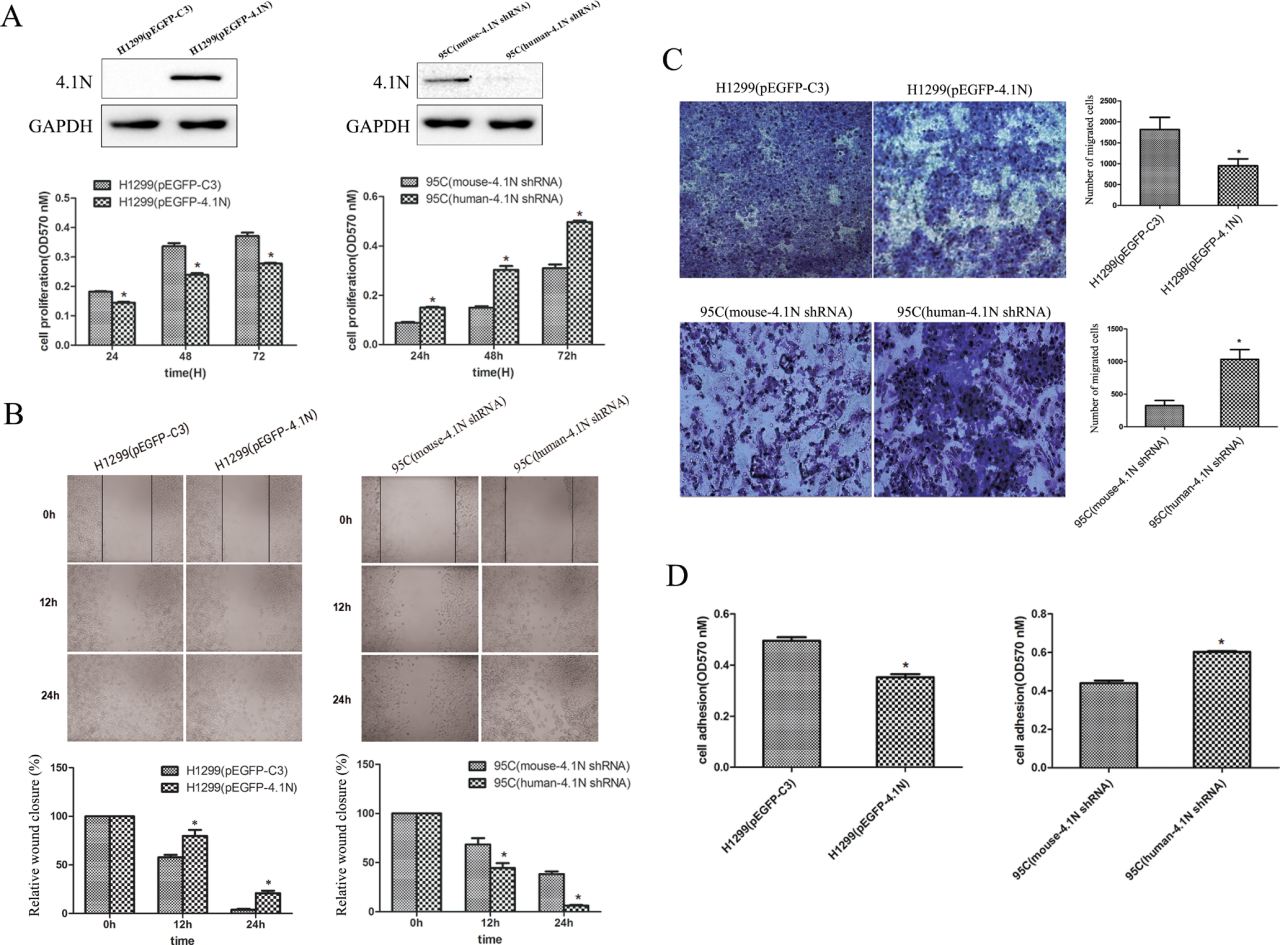
Anti-tumor effects of 4.1N on the proliferation, migration and adhesion in H1299 and 95C cells *in vitro* Both 95C /H1299 cells were transiently transfected with the same amount of human-4.1N shRNA/pEGFP-4.1N or with the negative control mouse-4.1N shRNA/null pEGFP vector. (**A**) Cell proliferation was measured by MTT assay for 24 h, 48 h and 72 h after cell plating. The degree of proliferation was reflected by the optical density (OD) value at 570 nm; the larger OD value indicated more active cell proliferation. The data are presented as the mean ± standard deviation (SD) from three independent experiments. Top panel: the effectiveness of the plasmids on 4.1N expression was evaluated by Western blotting analysis. (**B**) More than 90% of the confluent monolayer of transfected cells was scratched and imaged by light microscopy at three time points 0, 12, and 24 h. The degree of motility was shown as percent of wound closure as compare with the 0 h time point. (**C**) The migration of transfected H1299 and 95C cells was measured by using transwell chambers. Representative images of cells penetrating the chamber membrane were shown. The results are expressed as the average number of cells in five random microscopic fields ± SD of three independent experiments. (**D**) Transfected cells were seeded into 96-well plates that were pre-coated with fibronectin and incubated for 1 h at 37°C. Nonadherent cells were washed away, while the adherent cells were stained with crystal violet; the absorbance of the released crystal violet after extraction with SDS was quantified by spectrophotometry at 570 nm. The results are the mean ± SD from three independent experiments. *indicates *p* < 0.05 versus control based on the Student's *t*-test.

Given the low expression of 4.1N in H1299 cells and the relatively high expression in 95C cells, the exogenous 4.1N expression plasmid pEGFP-4.1N was transiently transfected into H1299 cells to increase 4.1N expression. Conversely, human-4.1N shRNA was transiently transfected into 95C cells to silence the expression of endogenous 4.1N. The effectiveness of the plasmids used for 4.1N knockdown or over-expression is shown in Figure 2A. The MTT results show that pEGFP-4.1N suppressed H1299 cell proliferation at different time points (24 h, 48 h and 72 h) after transfection when compared to the pEGFP-C3- transfected H1299 cells. On the contrary, human-4.1N shRNA promoted 95C cell proliferation when compared to the mouse-4.1N shRNA-transfected 95C cells (Figure 2A).

Metastasis cascade involves enhanced cell motility and changes in cell-extracellular matrix (ECM) adhesion [18]. The wound healing assay and transwell migration assay were performed to evaluate the cell motility ability. The results show that compared with the respective NC group, the over-expression of 4.1N markedly impaired the motility ability of H1299 cells. On the contrary, 4.1N-knockdown 95C cells demonstrated a significant increase in motility ability (Figure 2B and 2C). Adhesion to matrix is beneficial for metastatic cells to plant themselves in distant locations. Accordingly, the over-expression of 4.1N inhibited the adhesion of H1299 cells to fibronectin, whereas knockdown of 4.1N increased the adhesion of 95C cells to fibronectin (Figure 2D). Another 4.1N-specific shRNA (PLKO.1-shRNA-4.1N) was used in 95C cells to further conform the anti-tumor properties of 4.1N (Supplementary Figure S2). Moreover, given that β1 integrin is a major fibronectin-binding cell surface receptor and associated with a worse prognosis in NSCLC [19, 20], we therefore examined the expression change of β1 integrin in response to the knockdown or over-expression of 4.1N. The results show that β1 integrin expression was negatively regulated by 4.1N, suggesting that β1 integrin is potentially involved in 4.1N-mediated regulation of cell adhesion (shown in Figure 5A).

### Down-regulation of 4.1N promoted tumor growth and metastasis of 95C cells *in vivo*

**Figure 3:**
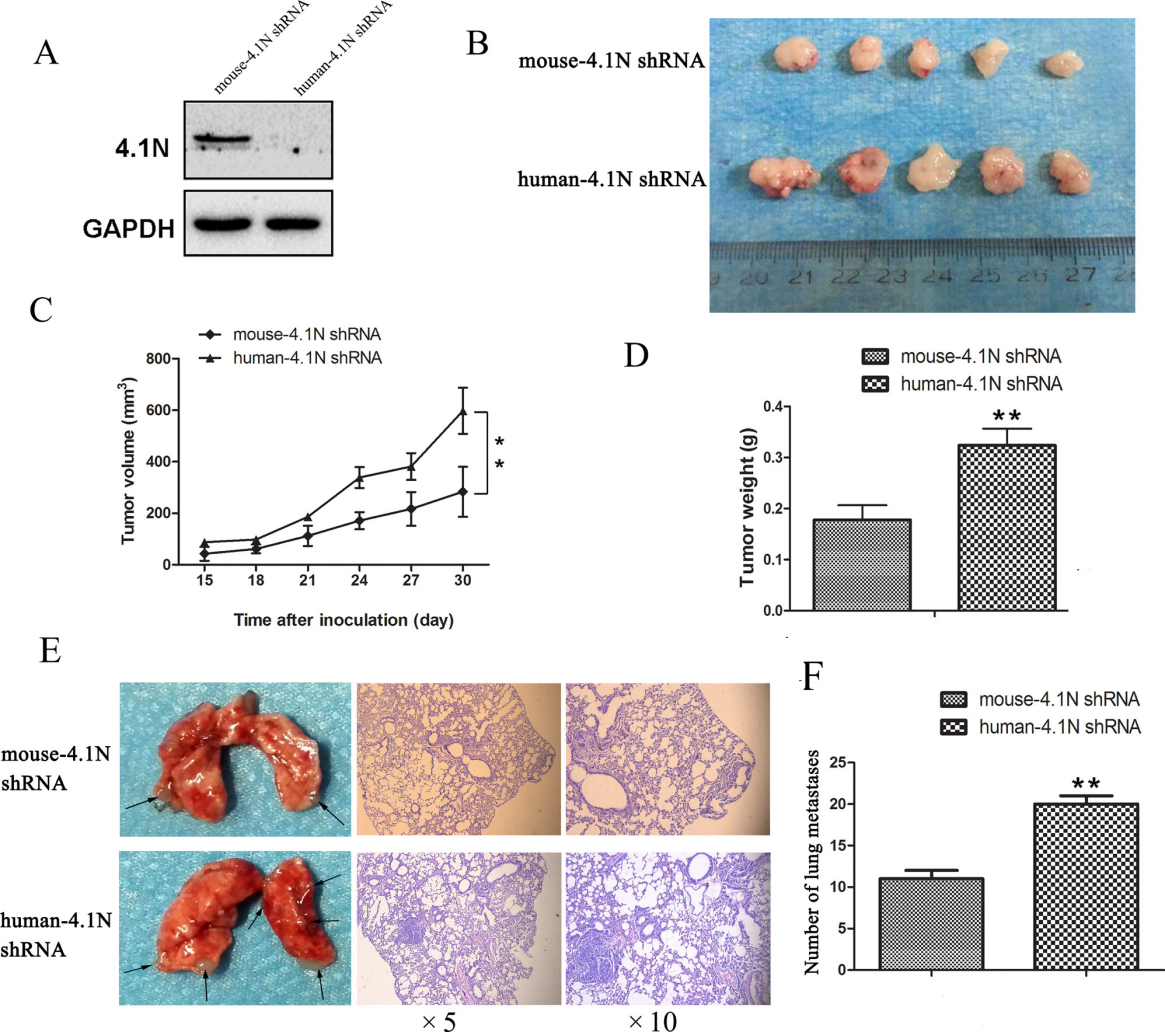
Anti-tumor effects of 4.1N were evaluated in the nude mice (**A**) Western blotting analysis confirmed that 4.1N was absent from 95C cells stably expressing human-4.1N shRNA. (**B**, **C** and **D**) Effects of 4.1N knockdown on tumor growth in the subcutaneous xenograft model. (B) Images of stripped tumors from mice (*n* = 5 mice for each group). (C) Tumor growth curves showed that 4.1N knockdown promoted the growth of xenografts in nude mice. The values at each time point are shown as mean tumor size (mm^3^) ± SEM (standard error of the mean). (D) The weight of xenograft tumors at the time of sacrifice. Values are shown as mean tumor weight (g) ± SEM. **p < 0.01 versus control. (**E** and **F**) Effects of 4.1N knockdown on tumor metastasis in the tail vein metastasis assay. (E) Gross examination of representative tumor-bearing lungs from the 4.1N knockdown and control groups (left). Representative H&E staining of lung sections (right). Magnification: × 5 and × 10. (F) Number of lung metastatic nodules based on observation of lung histological sections. Data are means±SD (*n* = 3 mice for each group). **p < 0.01.

To further assess the anti-tumor effects of 4.1N on cell growth and metastasis *in vivo*, the 95C cells stably expressing human-4.1N shRNA or mouse-4.1N shRNA were established (Figure 3A). Then, the cells were subcutaneously injected into the right subaxillary region of nude mice or intravenously injected into nude mice. The results show that tumors from the human-4.1N shRNA group grew faster than those from the NC group (Figure 3B and 3C). The average tumor weight of human-4.1N shRNA group (0.33 g) was obviously larger than that of NC group (0.18 g) (Figure 3D). Furthermore, the number of lung metastatic nodules was greater in mice injected with 95C cells/human-4.1N shRNA than in NC group (Figure 3E and 3F). These data indicate that 4.1N knockdown markedly enhanced 95C cell xenograft tumor growth and metastasis in mouse models.

### PP1 emerges as a novel 4.1N-interacting protein

**Figure 4:**
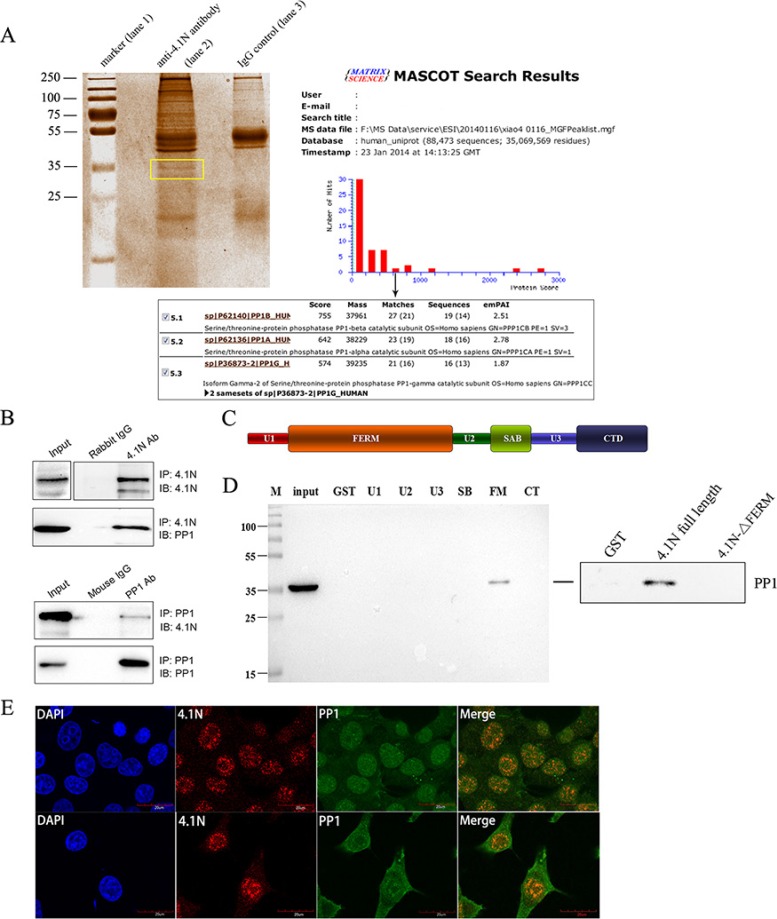
Interaction of 4.1N and PP1 in 95C cells (**A**) Coomassie blue-stained gel of immunoprecipitated proteins. Protein precipitates were immunoprecipitated with an anti-4.1N antibody (lane 2) and with the IgG negative control (lane 3). The differentially expressed band is indicated by the frame. The PP1 catalytic subunit, PP1 (α, β and γ2) was identified by the MASCOT search engine used to assess the data from LC-MS/MS by the identification of proteins from the Uniprot protein database. PP1 had the fifth-highest hit score among all possible hits that contained the sequences that matched the peptides within the sample. (**B**) 4.1N was immunoprecipitated from 95C cell lysates with an anti-4.1N antibody. 4.1N or PP1 in the immunoprecipitate was detected with an anti-4.1N or an anti-PP1 antibody. In contrast, PP1 was immunoprecipitated from 95C cell lysates with an anti-PP1 antibody. PP1 or 4.1N in the immunoprecipitate was detected with an anti-PP1 or an anti-4.1N antibody. (**C**) Schematic diagram showing the 4.1N protein domain organisation. 4.1N is composed of N-terminal FERM domain, an internal spectrin-actin-binding domain (SABD) and a C-terminal domain (CTD), which are separated by three unique regions (U1, U2 and U3). (**D**) Extracts from 95C cells were subjected to a pull-down assay with equivalent amounts of the GST and indicated GST-4.1N domain fusion proteins. PP1 binding was detected by immunoblotting with an anti-PP1 antibody. The endogenous PP1 in the extract was used as input. Right panel: Binding of PP1 to GST-tagged 4.1N full length proteins or GST-tagged 4.1N-ΔFERM fragments. Binding was assessed by pull-down assay, using anti-PP1 antibody for detection. (**E**) Subcellular co-localization of 4.1N and PP1 in 95C cells. Cells were co-stained for 4.1N (red) and PP1 (green). Nuclei were stained with DAPI (blue). The yellow/orange in the merged image indicates the co-localization of 4.1N and PP1. Scale bars: 20 μm.

To gain insight into the molecular mechanism of 4.1N, immunoprecipitation (IP) was used to determine the proteins that interact with 4.1N. After the precipitated protein complexes were separated on a 12% SDS-PAGE gel and stained with Coomassie blue, a differentially displayed band that encompassed proteins approximately 35 kDa in size was noted in comparison with the IgG control (Figure 4A). The band was excised, digested with trypsin, and subjected to LC-MS/MS (liquid chromatography tandem-mass spectrometry) analysis for protein identification. PP1 was identified as a potential 4.1N-interacting protein with a high hit score (Figure 4A). We further confirmed the interaction between 4.1N and PP1 by a Co-IP assay using 95C cell lysates. Endogenous PP1 was pulled down by an anti-4.1N antibody (Figure 4B). Similarly, endogenous 4.1N was also pulled down by an anti-PP1 antibody (Figure 4B).

To locate the 4.1N domain that is involved in the interaction, we performed a GST pull-down assay using various GST-tagged domains of 4.1N, GST-tagged full length 4.1N proteins and GST-tagged 4.1N fragments with a deletion of the FERM domain. The results showed that 4.1N associated with endogenous PP1 from 95C cell lysates mainly through its FERM (4.1-ezrin-radixin-moesin) domain while deletion of the whole 4.1N FERM domain abolished 4.1N interactions with PP1 (Figure 4C and 4D). Furthermore, double immunofluorescence staining for 4.1N and PP1 in 95C cells revealed that they were co-localized in both the cytoplasm and the nucleus (Figure 4E).

### 4.1N inhibits JNK-c-Jun signaling through PP1

**Figure 5:**
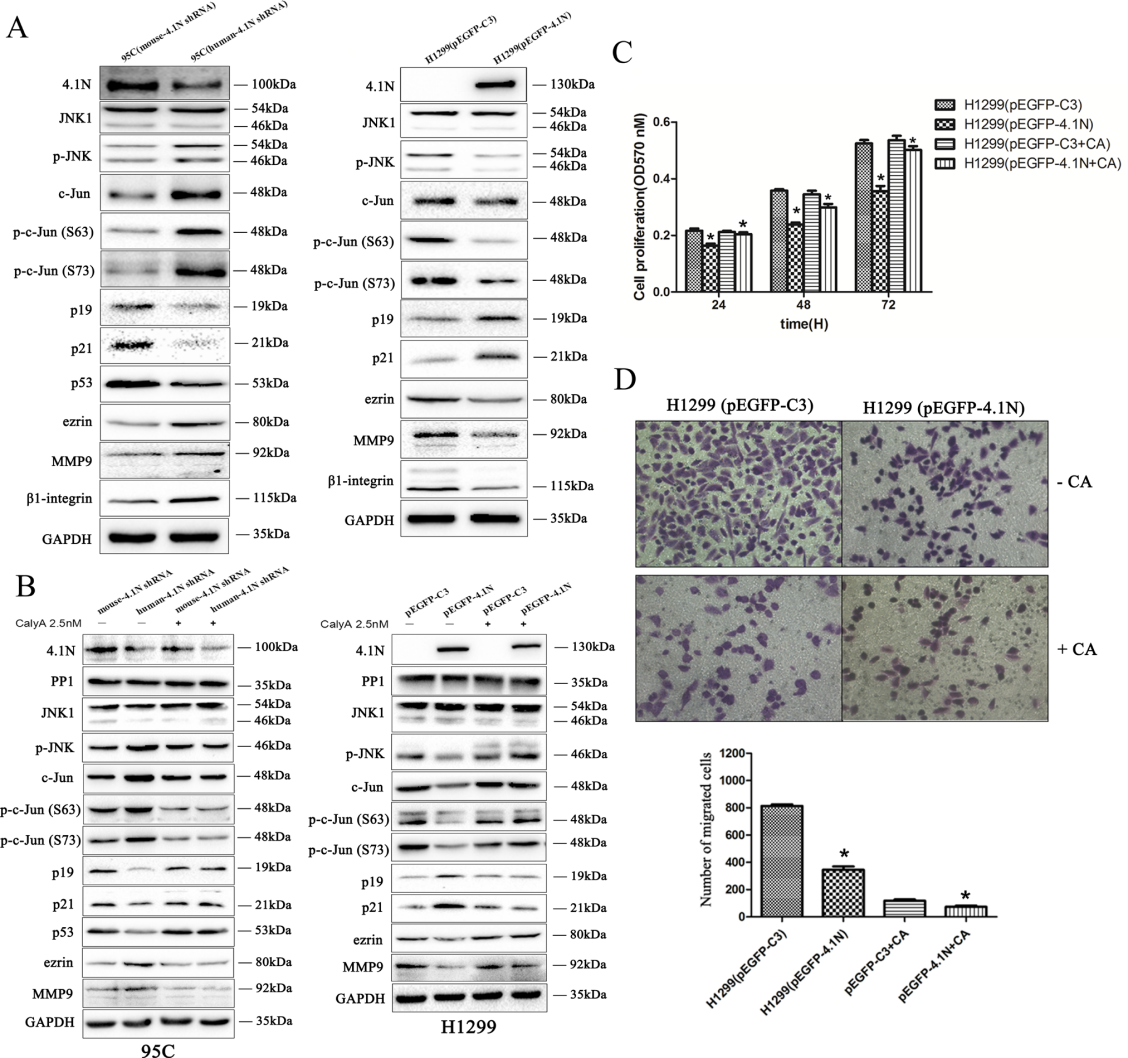
Effects of 4.1N on the expression of JNK-c-Jun signaling in NSCLC cell lines (**A**) Upon the change in 4.1N expression, expression analysis was performed of correlative JNK-c-Jun signaling molecules in H1299 and 95C cells by Western blotting. GAPDH served as the loading control. Moreover, Western blot analysis shows that β1 integrin expression was increased in 95C cells transfected with human-4.1N shRNA and was decreased in H1299 transfected with pEGFP-4.1N, compared with their counterparts. (**B**) PP1 inhibitor, calyculin A, abrogates the function of 4.1N in the negative regulation of the activity of JNK-c-Jun signaling. 48 h after transfection, H1299 and 95C cells were untreated (control) or pretreated with 2.5 nM of calyculin A for 1 h before harvesting the cell lysates; the expression of PP1 and correlative JNK-c-Jun signaling molecules were then detected by Western blotting. As H1299 is a p53-null cell line, we did not detect p53 expression in this cell line. (**C** and **D**) 48 h after transfection, H1299 cells were untreated (control) or pretreated with 2.5 nM of calyculin A for 1 h. Cells were then subjected to cell proliferation assays (C) and transwell migration assays (D) as described in the ‘Materials and methods’ section.

Considering that 4.1N knockdown propels cells toward a malignant phenotype, we further examined the activity of typical proto-oncogene ERK, AKT and JNK in response to 4.1N knockdown in 95C cells. Compared to ERK and AKT, JNK activity was the most severely impacted by 4.1N knockdown (Supplementary Figure S3). Given that JNK was found to be a substrate of PP1 [21], JNK-c-Jun pathway was selected for the further studies. Specifically, we found that, compared with the control group, down-regulation of 4.1N by 4.1N-shRNA in 95C cells markedly increased JNK phosphorylation, whereas the total JNK protein level remained unchanged. Consistently, the total c-Jun and phospho-c-Jun (Ser-63 and Ser-73) were significantly increased upon 4.1N knockdown. Furthermore, decreased 4.1N expression was accompanied by a significant decrease in the expression of p53, p21 and p19 that are potential downstream growth arrest effectors of c-Jun/AP-1 and by an increase in the expression of ezrin and MMP9 (matrix metalloproteinase 9) that are potential downstream metastasis-promoting effectors of c-Jun/AP-1 (Figure 5A). In contrast, reversed results (except for total JNK) were observed in 4.1N-overexpressing H1299 cells (Figure 5A). Correspondingly, the JNK inhibitor (SP600125) could keep p53, p21, p19 and ezrin from being regulated by 4.1N (Supplementary Figure S4). Therefore, 4.1N is able to negatively regulate the activity of JNK-c-Jun signaling pathway.

To test whether 4.1N inhibits JNK-c-Jun signaling through PP1, the changes of corresponding JNK-c-Jun signaling molecules were examined after treatment of cells with 2.5 nM calyculin A (a potent PP1 inhibitor). Our results show that the 4.1N-induced alteration of the JNK-c-Jun signaling molecules were completely eliminated by calyculin A in both H1299 and 95C cells as compared to their counterparts (Figure 5B, Supplementary Figure S5). Consistent with the molecular events, PP1 inhibition by calyculin A significantly weakened the inhibitory effects of 4.1N on growth and metastasis in H1299 cells (Figure 5C and 5D). The data thus support the hypothesis that 4.1N-mediated phenotypic changes in NSCLC cells and regulation of the JNK-c-Jun signaling at least partially through PP1.

### 4.1N positively regulates the phosphatase activity of PP1

**Figure 6:**
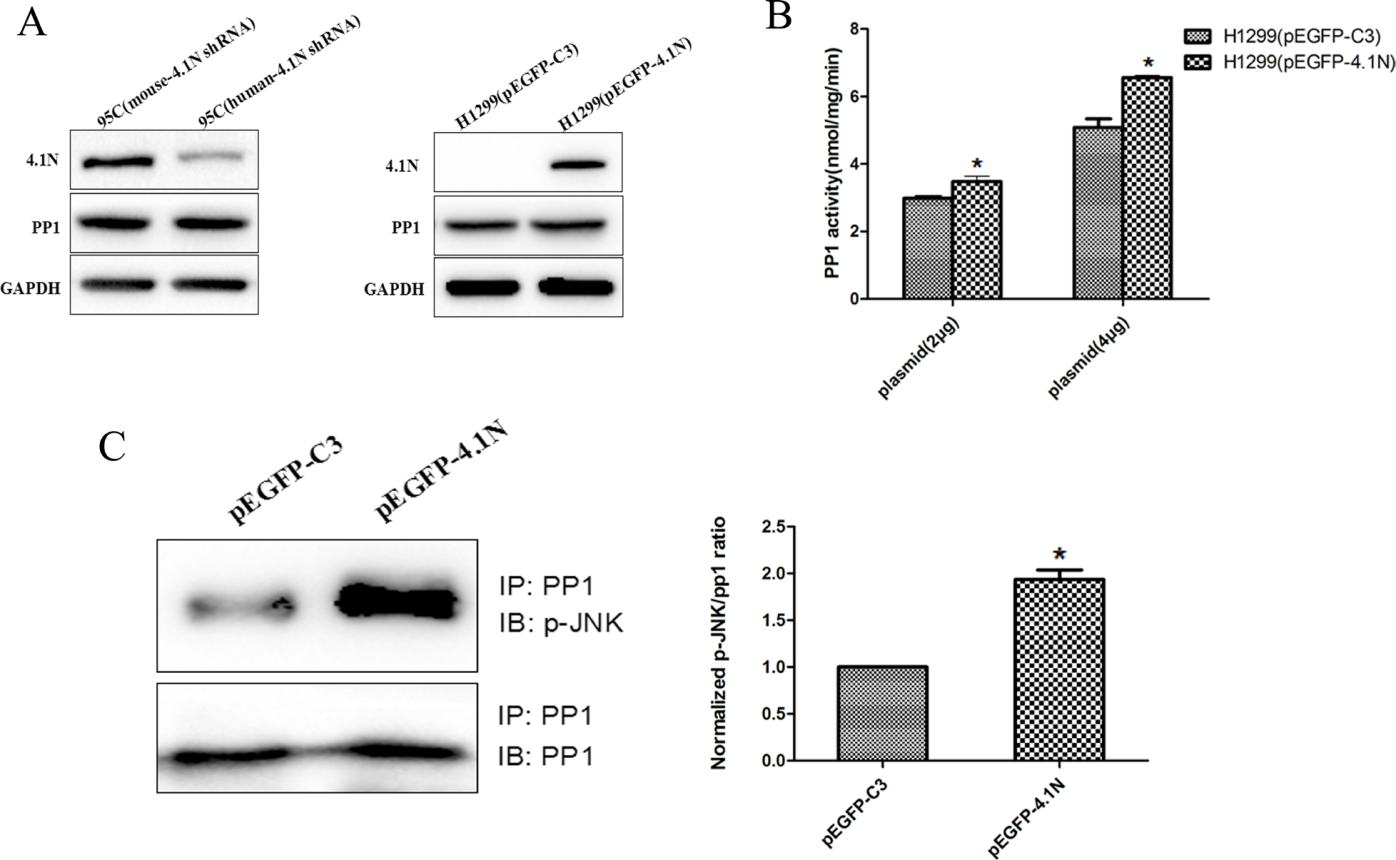
Effects of 4.1N on the expression and activity of PP1 in NSCLC cell lines (**A**) Western blotting analysis of PP1 expression in 95C cells transfected with human-4.1N shRNA or mouse-4.1N shRNA and in H1299 transfected with pEGFP-4.1N or pEGFP-C3. (**B**) The over-expression of 2 μg or 4 μg of pEGFP-4.1N in H1299 cells significantly increased (∼13% and ∼22%, respectively) the activity of PP1 activity. The data are shown as the mean ± SD from three independent experiments. (**C**) More p-JNK (normalized p-JNK/PP1 intensity was 1.9 ± 0.2) was detected in PP1 immunoprecipitates from H1299 cells that expressed pEGFP-4.1N as compared with the control (normalized p-JNK/PP1 intensity was 1). *p < 0.05.

Given that 4.1N interacts with PP1 and impact PP1 downstream JNK activity, we reasoned that 4.1N may directly regulate PP1 function. However, as shown in Figure 6A, the protein expression of PP1 remained unchanged upon altered expression of 4.1N. Therefore, we further examined whether 4.1N might regulate the phosphatase activity of PP1. Using [^32^P] phosphorylase α as substrate, PP1 activity in H1299 cells that transiently over-expressed 4.1N was significantly increased in a dose-dependent manner compared with the NC group (Figure 6B), which indicates that 4.1N is able to positively regulate PP1 activity.

We further investigated the interaction of p-JNK and PP1 in equivalent amounts of lysates from 4.1N-overexpressing H1299 cells and NC group by Co-IP. Quantitative analyses showed that the p-JNK: PP1 ratio for the amount of p-JNK in PP1 immune complexes from 4.1N-overexpressing H1299 cells was markedly higher than that from NC group. This increase in the association of PP1 with p-JNK may result in enhanced dephosphorylation of p-JNK by PP1 (Figure 6C).

## DISCUSSION

In this study, our data support that 4.1N behaves as a growth and metastasis-related tumor suppressor in NSCLC due to the low expression of 4.1N that was more prone to occur in metastatic NSCLC cells, poorly differentiated and advanced stage tumor specimens, and its anti-tumor effects *in vitro* and *in vivo*. We confirmed that 4.1N binds to PP1 through its highly conserved FERM domain. The FERM domain is required for localization or stability of 4.1 proteins at basolateral membrane in epithelial cells, while phosphorylation weakens the membrane- and cytoskeleton binding activity of FERM domain [22, 23]. Specially, 4.1N FERM domain harbors fifteen Ser and twelve Thr putative phosphorylation sites (predicted by KinasePhos2.0), which suggests that PP1-mediated dephosphorylation potentially contributes to the basolateral localization of 4.1N, thereby maintaining normal epithelial organization. Moreover, PP1 has recently been shown to be an essential part of a phosphatase relay controlling mitotic progression; this system is conserved in all eukaryotes. Therefore, there may be a role for 4.1N-PP1 interaction in mitotic control [24].

Emerging evidence suggests that sustained activation of JNK-c-Jun signaling is oncogenic in lung cancer, leading to promotion of tumor cell proliferation and survival ability, and to neoplastic transformation of bronchial epithelial cell lines [25–27]. JNK phosphorylation/activation has been detected in 45% (114/252) of NSCLC clinical specimens [27]. The absence of upstream phosphatases can lead to the constitutive activation of JNK. JNK is known to be dephosphorylated/inactivated directly by PP1. Although PP1 only dephosphorylates Thr^183^ of JNK, this seems to be sufficient for PP1 to dephosphorylate/inactivate JNK [21]. Here, 4.1N has been shown to prevent activation of JNK-c-Jun signaling, to positively regulate PP1 activity and to enhance the association of PP1 with p-JNK. In particular, inactivation of PP1 by the PP1 inhibitor calyculin A could diminish the inhibitory effect of 4.1N on JNK-c-Jun signaling and attenuate 4.1N-mediated tumor suppression. These results suggest that 4.1N as a scaffolding protein with no intrinsic phosphatase activity potentially inhibits JNK-c-Jun signaling through recruitment of PP1.

C-Jun is a main component of the AP-1 family [28, 29]. C-Jun/AP-1 mediates the proliferation regulatory effects of JNK-c-Jun pathway mainly through regulating transcription of cell cycle-related targets, such as p53, p21 and p19, all of which are typical negative cell cycle regulators [30]. p21 is a downstream target of p53 and mediates the anti-proliferative role of it [31]. p19 is able to protect p53 from degradation and transactivational silencing by sequestering Mdm2 [32]. In some case, p19 can inhibit cell growth independently of p53 [33]. C-Jun/AP-1 has been shown to bind to the promoter of p53, p21 and p19, and attenuate their transcription [34–37]. Our data show that 4.1N positively regulated the expression of p53, p21 and p19 in a JNK-c-Jun pathway-dependent manner. Therefore, 4.1N exerts anti-proliferative effects at least partially through these growth-inhibitory effectors.

C-Jun/AP-1 also directly stimulates effectors of metastasis such as ezrin and MMP9 through specifically binding to their promoters [38, 39]. Ezrin shares a sequence homology with the 4.1 proteins; however, it has been recognized as a pro-metastatic factor. High expression or activation of ezrin is highly implicated in the metastatic phenotype of various epithelial tumors [40, 41]. The opposite effects between ezrin and 4.1 proteins on tumor progression are likely caused by opposite effects of phosphorylation on their functions. Contrary to 4.1 proteins, phosphorylation activates ezrin, allowing ezrin to interact with transmembrane proteins and the cytoskeleton [40], while ezrin can be dephosphorylated by PP1 [15]. Interestingly, ezrin expression was negatively regulated by 4.1N in our study, which suggests that 4.1N naturally antagonize ezrin in terms of its expression and phosphorylation through promoting PP1-mediated dephosphorylation.

Additionally, both ERK and AKT activity were also enhanced upon 4.1N knockdown in 95C cells. Sustained activation of ERK and AKT drive the cell cycle process through the G1/S boundary, thereby promoting cell proliferation and survival [42, 43]. PP1 has been shown to associate with and dephosphorylate AKT in breast cancer cells [44], suggesting that 4.1N can also potentially prevent AKT activation via 4.1N-PP1 interaction. However, there is no evidence to show that ERK is a substrate of PP1. Specially, Chu et al. has described that PP1 was not able to dephosphorylate ERK2 but selectively dephosphorylate JNK1 and p38 MAP kinase [21]. Therefore, 4.1N may regulate ERK activity through other molecules rather than through PP1.

**Figure 7:**
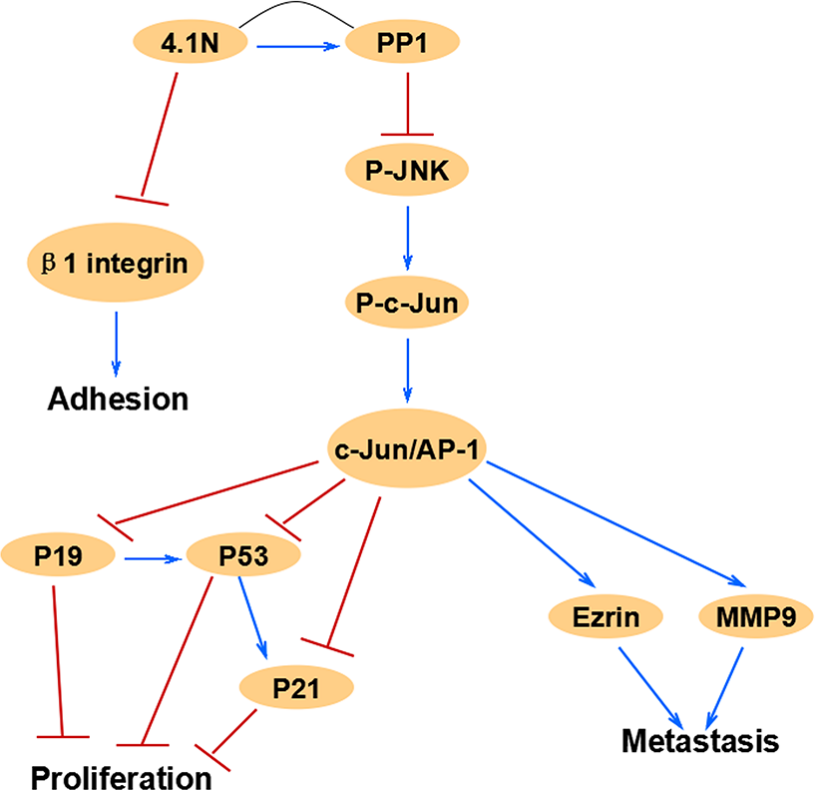
This schematic diagram summarizes the relationships of 4.1N and related JNK-c-Jun signaling molecules in this study, which are associated with cell proliferation, migration and adhesion

Altogether, our study strongly implies that 4.1N is a novel tumor suppressor in human NSCLC. Down-regulation of 4.1N expression is closely associated with the progression of NSCLC. Specially, 4.1N deficiency may be one of the factors that activate JNK-c-Jun pathway in NSCLC. Moreover, the mechanistic basis for 4.1N-mediated anti-tumor effects on cell proliferation, metastasis and adhesion likely involves the modulation of the cell cycle regulators p53, p21 and p19, the metastasis effectors ezrin and MMP9, and the adhesion receptor β1 integrin (Figure 7). Therefore, our study reveals a novel anti-tumor mechanism of 4.1N and suggests a potential 4.1N-targeted therapeutic strategy for NSCLC.

## MATERIALS AND METHODS

### Plasmids and reagents

Plasmid pEGFP-4.1N (human), pEGFP-C3, pENTR sh4.1N-human and pENTR sh4.1N-mouse were gifts from Dr. Xiuli An (New York Blood Center). Plasmid PLKO.1-empty vector and PLKO.1-shRNA-4.1N were purchased from Sigma-Aldrich. 4.1N shRNA target sequences as follows: pENTR sh4.1N-human, 5′-GCAACATCACTCGAAATAA-3′; pENTR sh4.1N-mouse, 5′-GCAACATCACTCTAGATAT-3′; PLKO.1-shRNA-4.1N, 5′-CCAGAAGATTGCCAAGAAATACTCGAGTATTTCTTGGCAATCTTCTGG-3′. PP1 inhibitor Calyculin A (Cell Signaling Technology), JNK inhibitor SP600125 (Selleckchem), X-tremeGENE HP DNA transfection reagent (Roche), PP1 phosphatase assay kit (GenMed).

### Cell lines and patient specimens

The NSCLC cell lines H1299, H460, SK-MES-1 and 95C were purchased from Cell Bank of Type Culture Collection of the Chinese Academy of Sciences, Shanghai Institute of Cell Biology. H1299 and H460 cells were cultured in RPMI 1640 medium (HyClone) supplemented with 10% fetal bovine serum (FBS, Invitrogen). SK-MES-1 and 95C cells were cultured in DMEM medium (HyClone) supplemented with 10% FBS. All cells were cultured in a humidified incubator at 37°C and 5% CO_2_.

NSCLC paraffin-embedded tissue samples collection were approved by the Ethic Committee of the Xiangya Hospital of Central South University. We randomly selected tissues from ninety-nine pairs of NSCLC surgical specimens, including 52 pairs of LAC samples, 46 pairs of LSCC samples, 1 pair of LCLC, and an additional 10 normal lung tissue specimens. Each tumor sample was located at least 2 cm away from the edge. The age of the cases ranged from 20 to 76 years, and the average age was 60 years. Histological grades were classified according to the WHO classification as well (grade I), moderately (grade II) and poorly differentiated (grade III). The TNM stage was performed according to the AJCC7 classification.

### Immunohistochemistry and evaluation of the results

The slides were deparaffinized in xylene and dehydrated through graded ethanol solutions and water. With respect to antigen retrieval, slides were heated in a microwave with citrate buffer (pH 6.0) for 15 minutes. After blocking the endogenous peroxidase activity with 3% H_2_O_2_ for 10 minutes, the slides were blocked with 10% normal goat serum for 30 minutes and then incubated with anti-4.1N HP antibody overnight at 4°C. Staining was visualized with diaminobenzidine (DAB; Sigma-Aldrich), which was placed onto the sections for 1 minute. After staining, sections were counterstained with hematoxylin. Then, the slides were washed with PBS, dehydrated, cleared in xylene and mounted with permount.

For evaluation, 4.1N staining was assessed by an addition of the cytoplasmic staining intensity and the percentage of positive tumor cells. The staining intensity was scored as 0 (negative), 1 (weak) or 2 (strong). The percentage of positive tumor cells was scored as 0 (negative), 1 (1–10%), 2 (11–40%), 3 (41–70%), or 4 (71–100%). A sample was defined as negative if the final score was 0–3 and as positive if the final score was 4–6. *P* values were calculated with the χ^2^ test.

### Cell transfection

Cells were seeded in six-well plates and were transfected with 2 μg/well of plasmids using the X-tremeGENE HP DNA transfection reagent, according to the manufacturer's instructions (Roche). 48 hours after the transfection, the cells were used for further experiments. To make stable cell lines deficient in 4.1N expression, the transfected cells were cultured in the selection-medium containing 400 μg/ml Zeocin. Three weeks later, survival colonies were isolated. The expression of 4.1N was detected by Western blotting. Then, the 4.1N-deficient clonal cells were further cultured under the selection-medium with 200 μg/ml Zeocin.

### Cell proliferation assay

The cell viability was evaluated by MTT assay. The transfected cells were seeded in triplicate in a 96-well plate at a density of 2 × 10^3^ cells per well. After 24 h, 48 h and 72 h, 100 μl of 5 mg/ml MTT in PBS was added to each well, and incubated for 4 h. Then, the formazan crystals were dissolved in 100 μl DMSO, and the absorbance was measured at a wavelength of 570 nm using a microplate reader. The experiment was repeated independently three times.

### Wound-healing assay

Transfected cells were grown on 60-mm plates. When the cell density reached 90% confluence, a linear wound was created by scraping the cell monolayer with a sterile P200 pipette tip. The floating cells were removed by washes with PBS, and then, adherent cells were incubated in fresh medium without serum. The healing process was imaged at 0 h, 12 h and 24 h. The wound closure was quantified by measuring the distance between the invading front of cells using Image J.

### Transwell migration assay

1 × 10^5^ transfected cells in 200 μl of medium containing 1% FBS were seeded into the upper chamber of wells (8 μm pore size; Corning Inc), and 0.7 ml of medium containing 10% FBS was added to the lower chamber. After 24 h incubation at 37°C and 5% CO_2_, the cells attached underneath the chamber membrane were fixed with 4% formaldehyde, stained with 0.1% crystal violet, and then photographed (10 × magnification). The average number of migrating cells was counted in at least five random microscopic fields.

### Cell adhesion assay

2 × 10^5^ transfected cells were seeded in 96-well plates that were coated with 10 μg/ml fibronectin (Becton Dickinson) and incubated at 37°C for 60 minutes. The cells were gently washed three times with PBS and fixed in 4% paraformaldehyde for 15 minutes. The cells were then stained with 0.2% crystal violet for 15 minutes. After a wash step and when the plates were dry, the crystal violet was dissolved with 2% SDS in PBS, and the absorbance was measured by spectrophotometry at 570 nm.

### Tumorigenicity assay in nude mice

The tumorigenic and metastatic properties of cells were evaluated by subcutaneous (SC) and intravenous (IV) inoculation of four to six-week-old athymic BALB/c nude mice (purchased from the animal center of Shanghai, China) with 95C cells lines stably expressing ectopic human-4.1N shRNA or mouse-4.1N shRNA. For SC inoculation, 3 × 10^6^ cells suspended in 200 μl serum-free DMEM medium were injected into the right subaxillary region of nude mice. After two weeks, all mice were regularly monitored every three days for tumor sizes. The tumor size was measured with a vernier caliper and calculated with the formula [V = (W^2^ × L)/2], where W and L are the shortest and longest diameter, respectively. Mice were sacrificed after two weeks of monitoring. The resected tumors were then photographed and weighed. For IV inoculation, mice were injected through the tail vein with 3 × 10^6^ cells in 200 μl serum-free DMEM medium. Mice were sacrificed seven weeks after injection, and metastatic nodules on the surface of lungs were photographed. Then, lung tissues were fixed with 10% formalin, embedded in paraffin, sliced into 5–7 μm sections, and stained with hematoxylin and eosin (H&E) for light microscopy examination.

### Western blotting

Cells were lysed with RIPA buffer (150 mM NaCl, 25 mM Tris-HCl pH 7.4, 0.1% SDS, 1% Triton X-100, 1% deoxycholate, 2 mM EDTA) that contained a protease or phosphatase inhibitor mixture (Roche). 50 μg proteins were separated by SDS-PAGE gels, transferred onto nitrocellulose membranes, and incubated with primary antibodies. After incubation with appropriate HRP-conjugated secondary antibodies, signals were detected by ECL HRP substrate (Advansta). The following primary antibodies were used: Anti-human 4.1N HP (U1) domain (New York Blood Center); JNK1 (Santa Cruz, sc-1648), phospho (Thr-183 and Tyr-185)-JNK (Bioworld, BS4322), c-Jun (Santa Cruz, sc-44), PP1 (Santa Cruz, sc-7482), p53 (Cell Signaling Technology, #9282), p21 (Santa Cruz, sc-397), p19 (Santa Cruz, sc-65594), phospho (Ser-63/73)-c-Jun (Bioworld, BS4045/BS4046), GAPDH (Santa Cruz, sc-25778), ezrin (Bioworld, BS1118), MMP9 (Bioworld, BS1241), β1 integrin (Abcam, ab30394), phospho-ERK (Santa Cruz, sc-7383), ERK1/2 (Santa Cruz, sc-94), AKT1 (Santa Cruz, sc-5298) and phospho-AKT (ser473) (Cell Signaling Technology, #9271).

### Immunofluorescence

Cells were grown on coverslips and fixed in 4% paraformaldehyde in PBS for 15 minutes at room temperature. After three washes in PBS, the cells were permeabilized with 0.2% Triton X-100 in PBS for 5 minutes. The cells were then blocked in 5% BSA for 1 h and incubated with primary antibodies to 4.1N and PP1 at 4°C overnight. Next, the cells were incubated with Alexa Fluor 594- and 488-conjugated secondary antibodies (Abcam) for 1 h at room temperature. The nuclei were stained with DAPI (0.6 mg/ml; Sigma). The stained cells were visualized and imaged with a Nikon Eclipse E800 M epifluorescence microscope.

### Purification of the 4.1N-interacting protein complex

95C cells were lysed in IP buffer (0.1% NP–40, 420 mM KCl, 50 mM HEPES buffer pH 8.3, 1 mM EDTA) at 4°C for 30 minutes. A total of 1 mg of cell lysate was incubated with 2 μg anti-4.1N antibody or IgG control (Santa Cruz) at 4°C overnight with rotation, followed by the addition of 30 μl protein G-coupled magnetic beads (Life Technologies) with rotation at 4°C for 2 h. The beads were collected magnetically and washed four times with IP buffer. The immunoprecipitated proteins were then separated by 12% SDS-PAGE after the addition of SDS sample buffer and 5 minutes of boiling. Protein bands were visualized by Coomassie blue and were excised for further protein identification by LC-MS/MS analysis.

### Co-IP

Cells were lysed with IP buffer at 4°C for 30 minutes. A total of 1 mg of cell lysate was incubated with 2 μg anti-4.1N antibody, 2 μg anti-PP1 antibody or 2 μg IgG at 4°C overnight with rotation. The immunoprecipitates were isolated with 30 μl protein G beads, were then separated by SDS-PAGE and finally analyzed by Western blotting.

### GST pull-down assay

Recombinant GST-4.1N domain proteins (GST-U1/-U2/-U3/-FERM/-SAB/-CTD) from the isopropyl β-D-1-thiogalactopyranoside (IPTG)-inducible prokaryotic expression vector pET42a were over-expressed and purified from the *Escherichia coli* BL21 cells. Cell lysates from 95C cells were incubated for 1 h at 4°C with equivalent amounts of recombinant GST-4.1N domain proteins or GST proteins that were immobilized on MagneGST-glutathione beads (Promega). After extensive washes with TENT buffer (1% TritonX-100, 140 mM NaCl, 2 mM EDTA, and 20 mM Tris pH 8.0), the beads were boiled for 5 minutes in SDS sample buffer. The bound proteins were then subjected to SDS-PAGE and Western blotting. GST-alone proteins were used as negative control in this assay.

### 4.1N cDNA cloning

Full length 4.1N cDNA was amplified from plasmid pEGFP-4.1N by PCR using Platinum Taq DNA polymerase high fidelity (Thermo Fisher Scientific) with the following primer pairs: 5′-GGATATCGGGGATCCGAATTCATGACAACAGAGACAGGTCCCG-3′ and 5′-GTGGTGGTGGTGGTGCTCGAGTCAGGATTCCTGTGGCTTCTTG-3′. The PCR products containing an 5′ EcoRI and 3′ XhoI restriction site were cloned into pET-42a vectors, using the ClonExpress II One Step Cloning Kit (Vazyme Biotech Co., Nanjing, China).

The ΔFERM construct was made by deleting the FERM sequence from the pET-42a-4.1N vectors using Mut ExpressMultiS Fast Mutagenesis Kit V2 (Vazyme Biotech Co., Nanjing, China) according to the manufacturer's instructions, with the following primer pairs: 5′-GCGGCATATGGCACTCTTGAACTTCTTGGCAATCTTCTGA-3′ and 5′-AAGAGTGCCAT ATGCCGCGTGTCACCGGAGCCCCCACCCA-3′. The pET-42a-4.1N-ΔFERM cyclization constructs were obtained by using ClonExpress II One Step Cloning Kit. Nucleotide sequences of all cDNA clones were verified by restriction endonuclease analysis and DNA sequencing.

### Phosphatase activity assay

Phosphatase activity of PP1 was determined by the measurement of ^32^P released from [^32^P] phosphorylase α at 30°C in the presence of fostriecin (a PP2A inhibitor; Sigma) using a PP1 phosphatase assay kit (GenMed) according to the manufacturer's protocol. One unit of phosphatase activity is defined as the release of 1 nmol of ^32^P/mg/min from the substrate.

### Statistical analyses

All statistical analyses were performed with the SPSS 16.0 statistical software package. Student's *t*-test was used to determine the significance of the differences between the control and the experimental groups. The chi-square test was used to analyze the relationship between 4.1N expression and the clinicopathologic features. Error bars were used to indicate the standard deviation of the data, and *P* < 0.05 was considered statistically significant.

## SUPPLEMENTARY MATERIALS FIGURES


