# Correction: Protein 4.1N acts as a potential tumor suppressor linking PP1 to JNK-c-Jun pathway regulation in NSCLC

**DOI:** 10.18632/oncotarget.27282

**Published:** 2019-10-22

**Authors:** Zi Wang, Bianyin Ma, Hui Li, Xiaojuan Xiao, Weihua Zhou, Feng Liu, Bin Zhang, Min Zhu, Qin Yang, Yayue Zeng, Yang Sun, Shuming Sun, Yanpeng Wang, Yibin Zhang, Haibo Weng, Lixiang Chen, Mao Ye, Xiuli An, Jing Liu

**Affiliations:** ^1^ The State Key Laboratory of Medical Genetics and School of Life Sciences, Central South University, Changsha, China; ^2^ Department of Medicine, University of California, Irvine, CA, USA; ^3^ Department of Biochemistry, College of Medicine, Jishou University, Jishou, China; ^4^ Department of Histology and Embryology, Xiangya School of Medicine, Central South University, Changsha, China; ^5^ Molecular Science and Biomedicine Laboratory, State Key Laboratory for Chemo/Biosensing and Chemometrics, College of Biology, College of Chemistry and Chemical Engineering, Collaborative Innovation Center for Chemistry and Molecular Medicine, Hunan University, Changsha, China; ^6^ College of Life Sciences, Zhengzhou University, Zhengzhou, China; ^7^ Laboratory of Membrane Biology, New York Blood Center, New York, NY, USA

**This article has been corrected:** During the assembly of Figure 2B, the same image was inadvertently used for both H1299 (pEGFP-C3) and H1299 (pEGFP-4.1N) groups at 0h time point. The proper Figure 2 B specific for H1299 (pEGFP-C3) and H1299 (pEGFP-4.1N) groups are shown below. The authors declare that these corrections do not change the results or conclusions of this paper.

**Figure 2:**
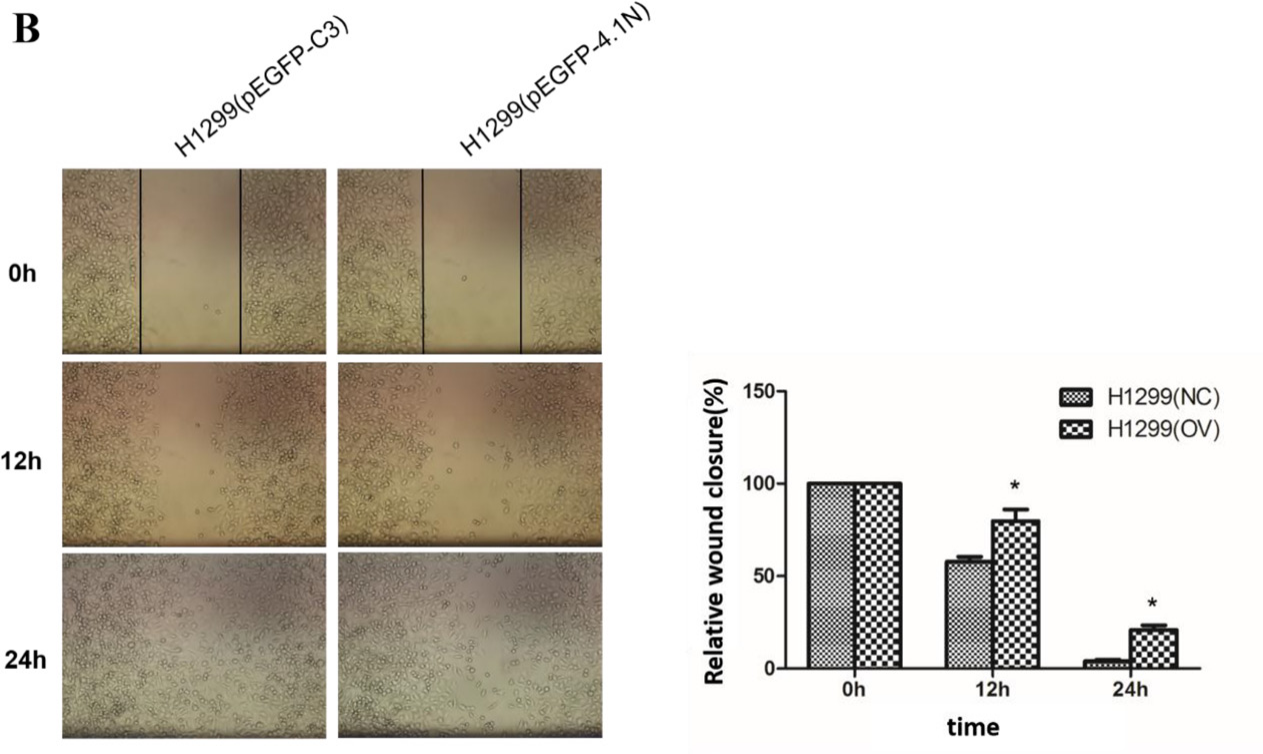
Anti-tumor effects of 4.1N on the proliferation, migration and adhesion in H1299 and 95C cells *in vitro*. Both 95C /H1299 cells were transiently transfected with the same amount of human-4.1N shRNA/ pEGFP-4.1N or with the negative control mouse-4.1N shRNA/ null pEGFP vector. (**B**) More than 90% of the confluent monolayer of transfected cells was scratched and imaged by light microscopy at three time points 0, 12, and 24 h. The degree of motility was shown as percent of wound closure as compare with the 0h time point. The results are the mean ± SD from three independent experiments. Representative images were shown. *indicates *p* < 0.05 versus control based on the Student’s t-test.

Original article: Oncotarget. 2016; 7:509–523. 509-523. https://doi.org/10.18632/oncotarget.6312

