# Supplementary material for: Protein 4.1N acts as a potential tumor suppressor linking PP1 to JNK-c-Jun pathway regulation in NSCLC
Source: Oncotarget. 2015 Nov 13;7(1):509–23. doi: 10.18632/oncotarget.6312 (PMC4808014; doi:10.18632/oncotarget.6312)
Supplement: Supplementary file 1 [file oncotarget-07-0509-s001.pdf]

Protein 4.1N acts as a potential tumor suppressor linking PP1 to JNK-c-Jun pathway regulation in NSCLC

Supplementary Materials

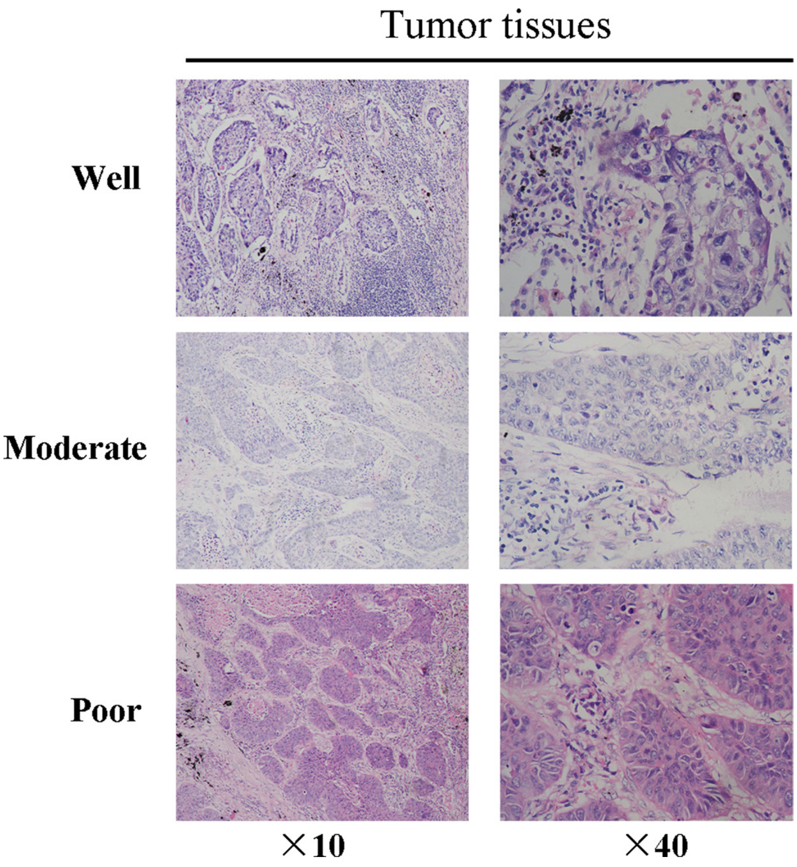

Supplementary Figure S1: Corresponding hematoxylin and eosin (H&E) staining of typical tumor tissue samples in the figure 1. Original magnification: x10 and x40.

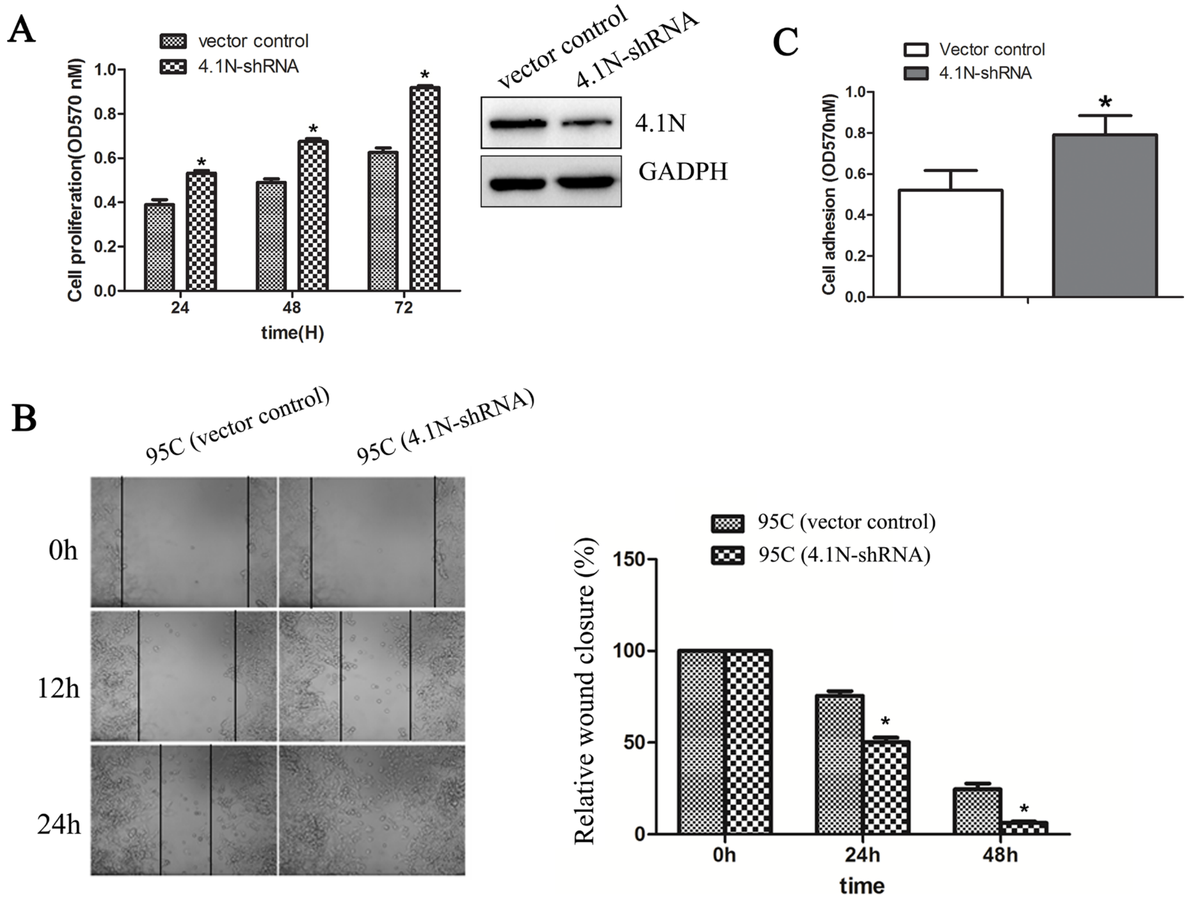

Supplementary Figure S2: Inhibition of 4.1N by the PLKO.1-shRNA-4.1N enhanced cell proliferation, migration and adhesion in 95C cells *in vitro*. 95C cells were transiently transfected with 2 μg of PLKO.1-shRNA-4.1N or PLKO.1-empty vector (control shRNA) for 48 h. Cells were then subjected to the MTT assay (**A**), wound-healing assay **(B)** and cell adhesion assay (**C**) as described in the 'Materials and methods' section. The results are the mean ± SD from three independent experiments. $*p < 0.05$.

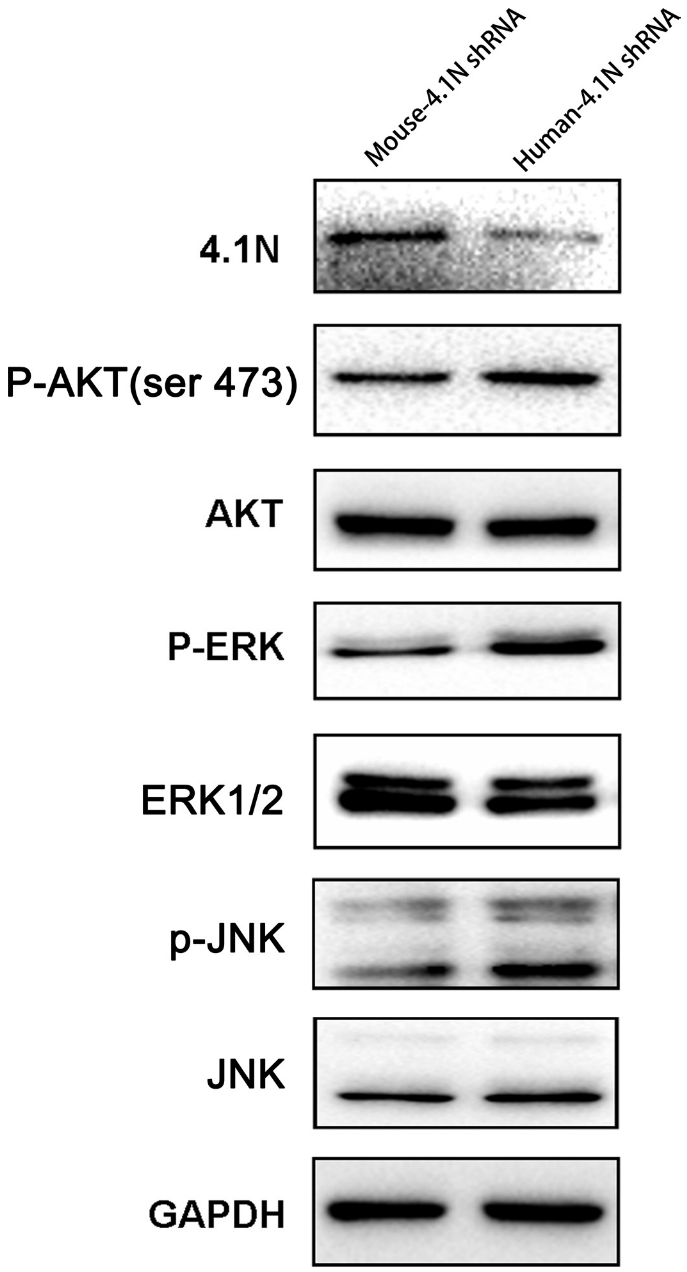

Supplementary Figure S3: Western blotting analysis of AKT, p-AKT, ERK, p-ERK, p-JNK and JNK expression in transiently transfected 95C cells.

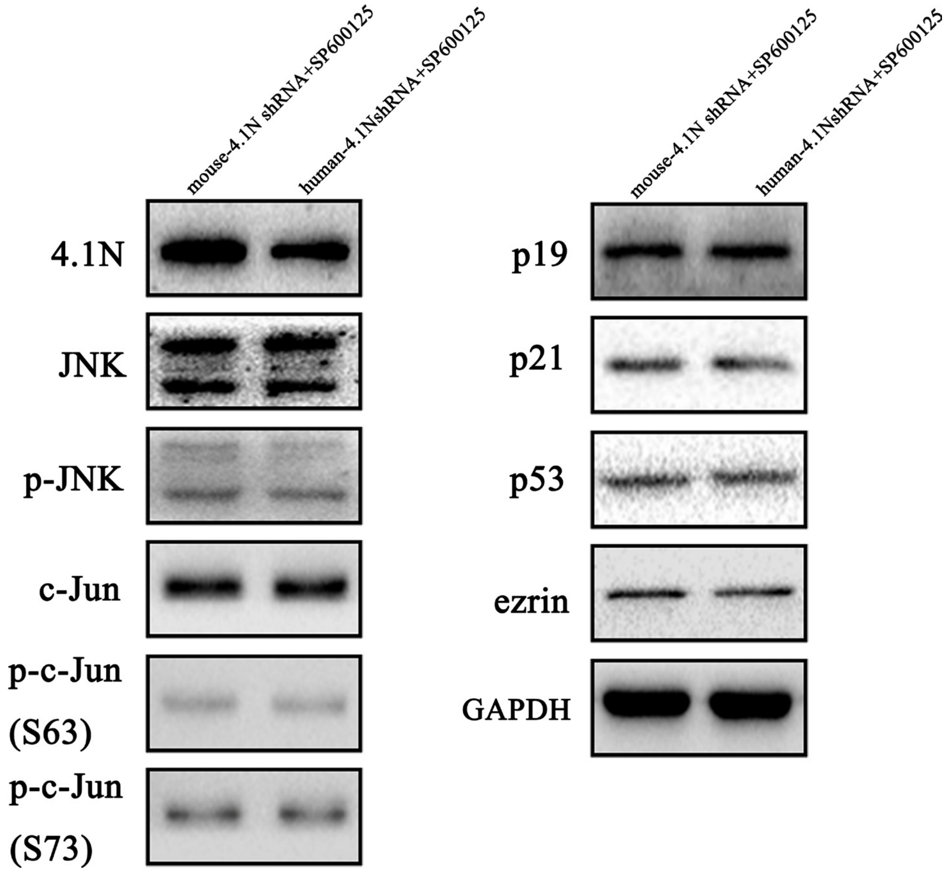

Supplementary Figure S4: Treatment with SP600125 (JNK inhibitor) prevented the activating effects of 4.1N knockdown on JNK-c-Jun signaling in 95C cells. 95C cells transfected with human-or mouse- 4.1N shRNA were treated with 50 μM of SP600125 for 2 h and then subjected to Western blotting analysis to examine related JNK-c-Jun signal molecules. 4.1N knockdown-induced the activation of c-Jun and ezrin, and the inactivation of p53, p21 and p19 were eliminated by SP600125 as compared with the counterpart.

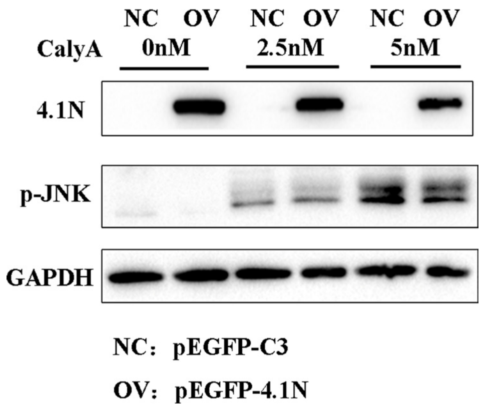

Supplementary Figure S5: PP1 inhibitor CalyA induced p-JNK increase in a dose-dependent manner. The pEGFP-4.1N or pEGFP-C3 transfected H1299 cells were treated with 0 nM, 2.5 nM or 5 nM calyculin A for 1 h, respectively.
